# Supplementary material for: Epithelioid Pleural Mesothelioma Is Characterized by Tertiary Lymphoid Structures in Long Survivors: Results from the MATCH Study
Source: Int J Mol Sci. 2022 May 21;23(10):5786. doi: 10.3390/ijms23105786 (PMC9144737; doi:10.3390/ijms23105786)
Supplement: Supplementary file 1 [file ijms-23-05786-s001.zip › ijms-1717159-supplementary/SupplementaryFigures.pdf]

Supplementary Materials

Epithelioid Pleural Mesothelioma Is Characterized by Tertiary Lymphoid Structures in Long Survivors: Results from the MATCH Study

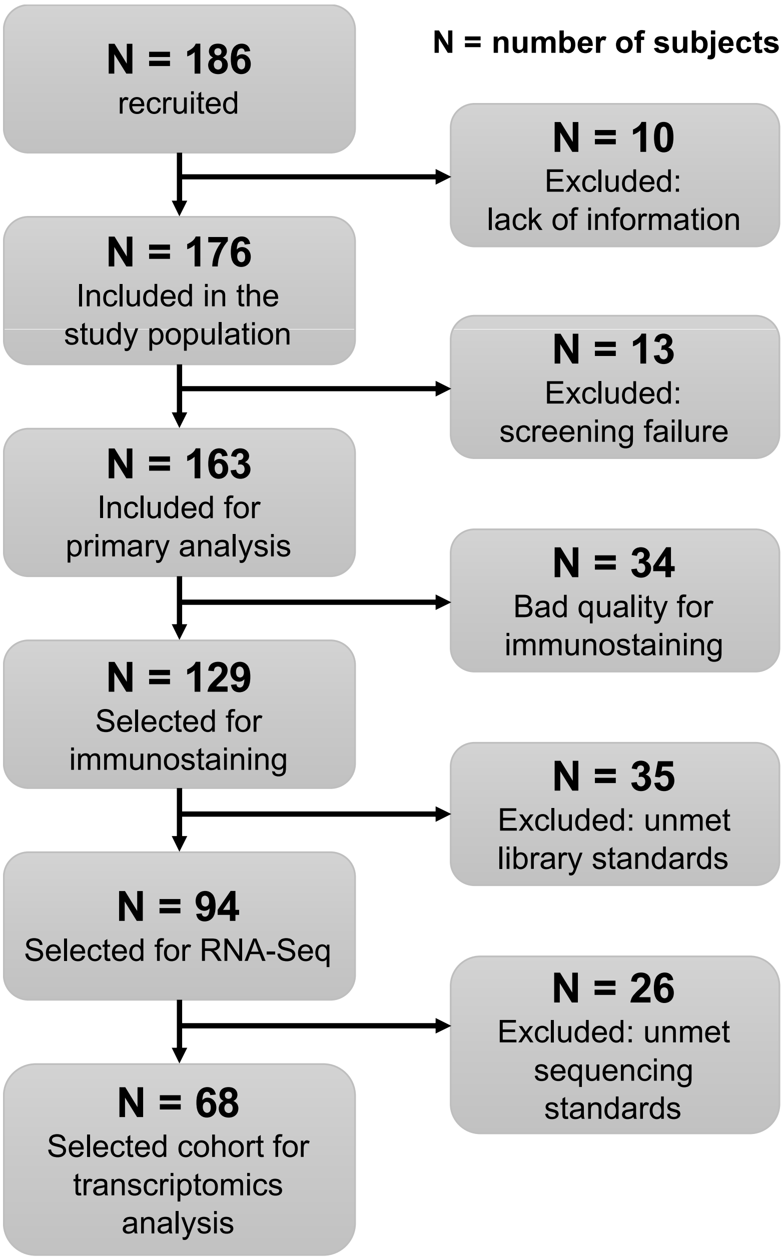

Figure S1. Flowchart representing the number of subjects recruited at the beginning of the study and the number of cases removed at each steps of the work.

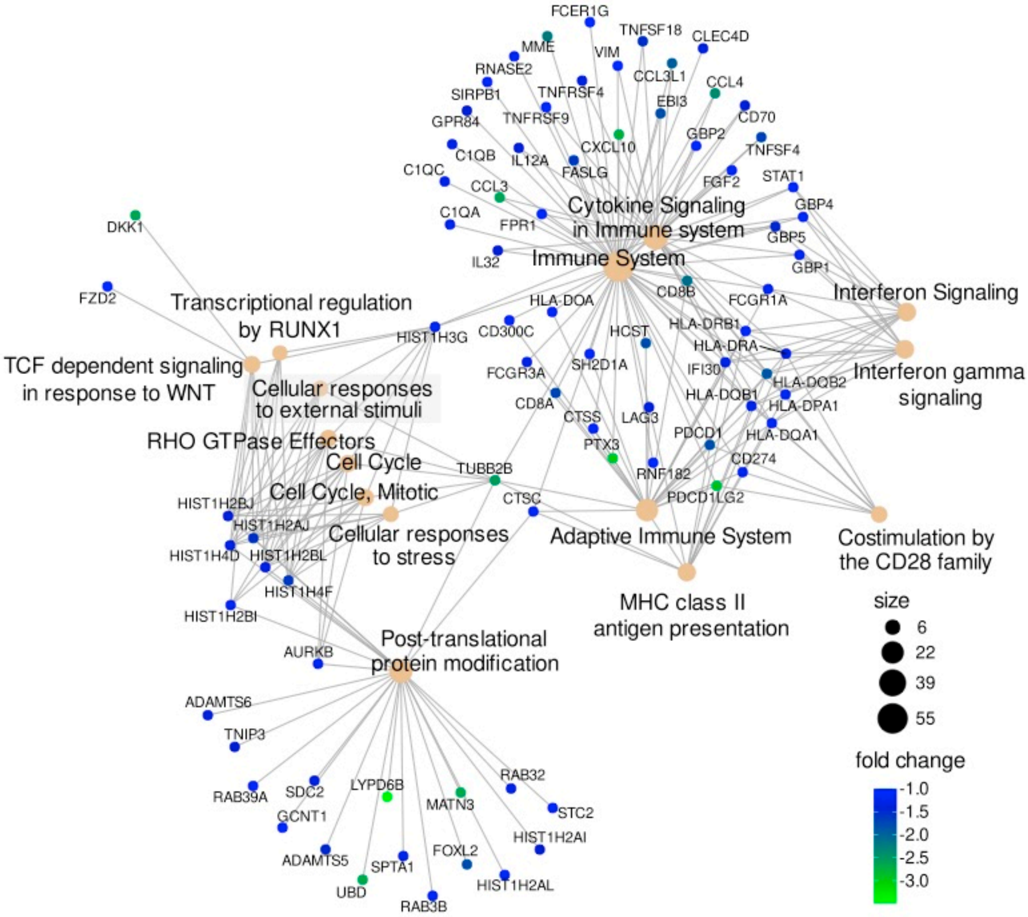

Figure S2. Map of the GSEA on DEGs in the comparison between non-ePM and eMP. Pathways are indicated with brown dots. Genes are colored based on their fold change as indicated in the legend. Edges connect genes to the pathway they belong to.
